# Polyarteritis Nodosa Associated With Hepatitis C Virus Infection

**DOI:** 10.7759/cureus.44129

**Published:** 2023-08-25

**Authors:** Shayan Amini, Zunirah Ahmed, Tamneet Basra, David Victor, Lillian Gaber, Sudha Kodali

**Affiliations:** 1 Internal Medicine, Houston Methodist Hospital, Houston, USA; 2 Gastroenterology, Methodist Health System, Houston, USA; 3 Hepatology and Transplant Medicine, Houston Methodist Hospital, Houston, USA; 4 Pathology, Houston Methodist Hospital, Houston, USA

**Keywords:** cryoglobulinemia vasculitis, hepatitis b infection, : acute kidney injury, polyarteritis nodosum, hepatitis c (hcv) infection

## Abstract

Polyarteritis nodosa (PAN) is a rare necrotizing vasculitis that affects medium-sized arteries. The association of hepatitis B virus (HBV)and HIV with PAN is well documented. Although there are documented cases of PAN in patients with hepatitis C virus (HCV) infection, the connection between PAN and HCV is not well established. We report a case of PAN in a patient with HCV infection who failed treatment with interferon.

## Introduction

Polyarteritis nodosa (PAN) is a necrotizing vasculitis that affects up to nine persons per million every year [1]. Prior to the development of the hepatitis B virus (HBV) vaccine, 30% of PAN cases were associated with HBV infection [2,3]. Despite its well-documented association with mixed cryoglobulinemia (MC), hepatitis C virus (HCV) infection has also been reported in patients with PAN. In their cohort of 161 patients with HCV, Saadoun et al. found that 19.3% had HCV-associated PAN [4]. Some of the common manifestations of PAN in HCV infection include purpura, arthralgia, weight loss, and multiplex mononeuritis [1]. HCV-PAN tends to occur in older patients and has a higher incidence in women [4]. Distinguishing PAN from MC is often challenging because circulating cryoglobulins are found in 40-60% of HCV patients [5].

## Case presentation

**Figure 1:**
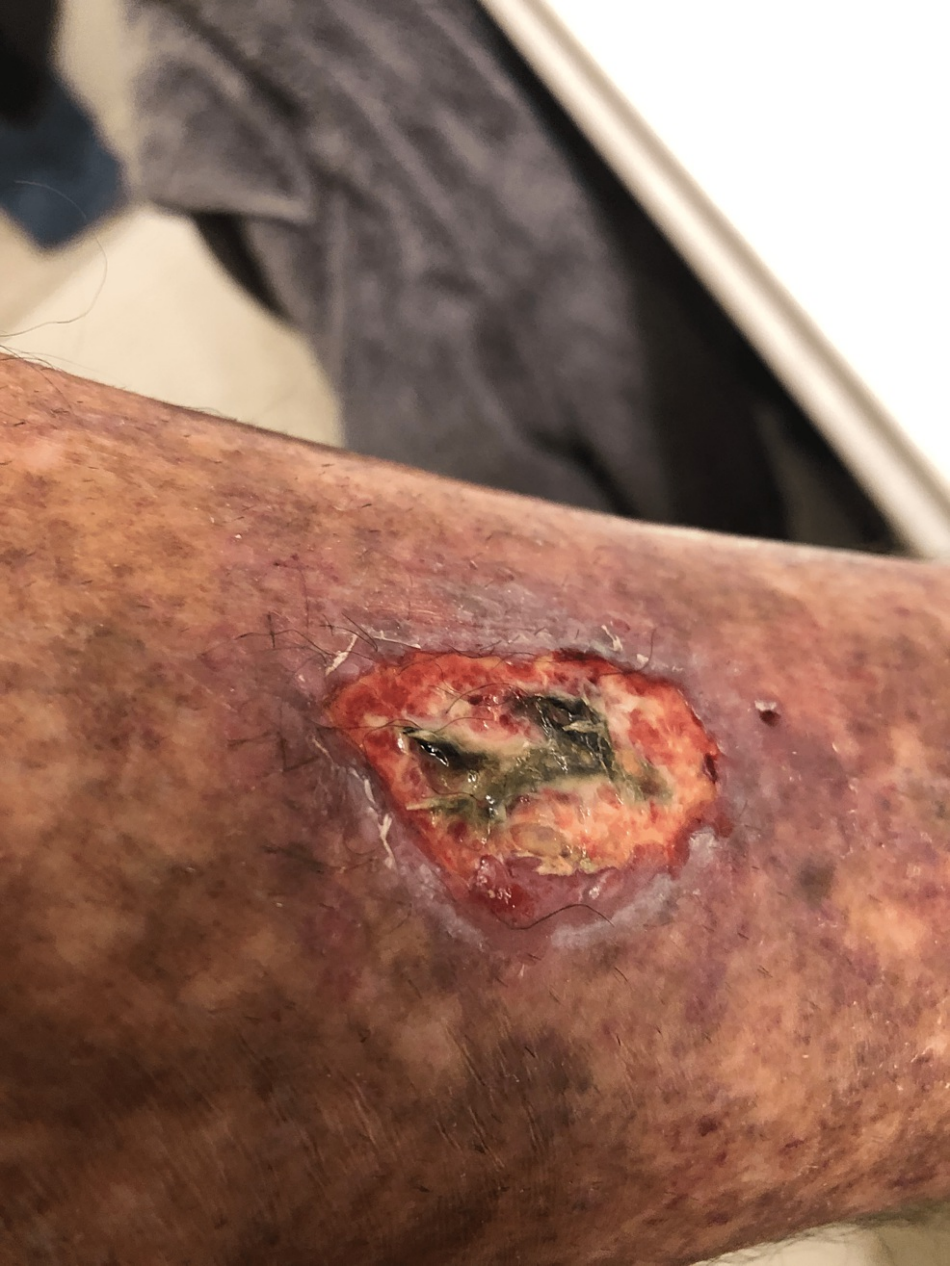
Lower extremity ulcerated wound

A 66-year-old male with a past medical history of HCV infection (diagnosed more than 30 years ago), diverticulitis, and dry gangrene of the left hand’s third digit for which he needed amputation presented with a two-day history of nausea, vomiting, poor appetite, and bilateral lower extremity ulceration without edema (Figure 1).

**Figure 2:**
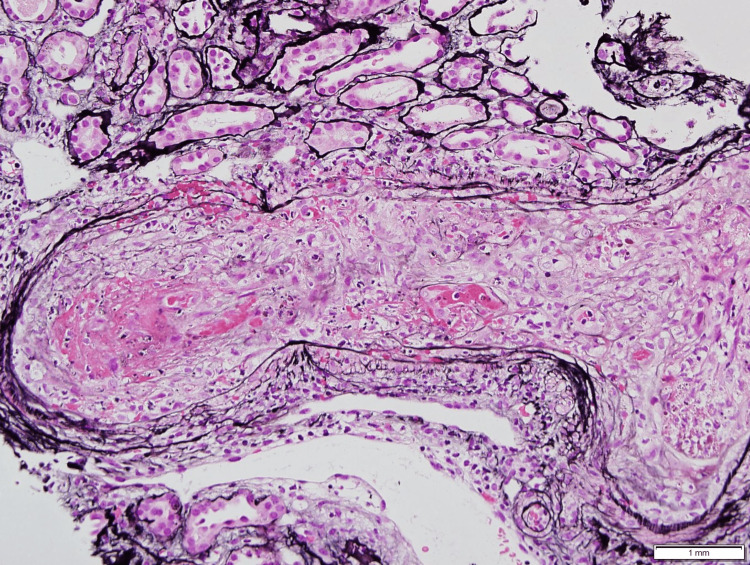
Renal biopsy The silver-stained section shows necrotizing inflammation of the medium-sized artery and its tributary with complete lumen obliteration and focal transmural inflammation. Several arterial cross-sections were similarly involved

The patient also reported a 15-20 pounds weight loss within the past month. The patient was recently seen by his hematologist and was instructed to go to a nearby hospital for further evaluation and possible plasmapheresis due to concern for MC. For his HCV infection, he was treated with interferon 30 years ago and was told that the HCV was in a dormant state and did not have a regular follow-up. However, the patient’s viral load was >1 million copies/ml two months prior to admission with an unremarkable hepatic ultrasound. On presentation, the patient was found to have an acute kidney injury with a serum creatinine of 2.04. Urinalysis showed 1+ protein, hematuria, and granular casts. Renal ultrasound was unremarkable. Autoimmune workup hinted at MC given elevated rheumatoid factor (225 IU/mL) and lower level of C4 (<2 mg/dL) compared to C3 (60 mg/dL). However, the coagulation workup showed normal cryoglobulin, cryofibrinogen, cryocrit globulin, and cryocrit fibrinogen levels. HCV polymerase chain reaction (PCR) was positive with a viral load of >1.3 million copies/ml. HBV core antigen was positive with negative HBV surface antigen and negative HBV PCR indicating prior exposure and spontaneous clearance. Due to concern for severe vasculitis contributing to acute renal failure, rheumatology was consulted and recommended prednisone 30 mg daily. A renal biopsy was done that showed necrotizing inflammation of medium-sized arteries consistent with PAN (Figure 2).

Before initiation of cyclophosphamide for PAN, HCV therapy with sofosbuvir-velpatasvir was started for active HCV infection along with entecavir for HBV prophylaxis given concern for an acute flare and reactivation, respectively. The patient received one round of cyclophosphamide 900 mg infusion and was continued on prednisone 30 mg daily. Unfortunately, the patient’s renal function continued to deteriorate necessitating the initiation of renal replacement therapy. The patient was eventually discharged home on prednisone 30 mg daily with a plan to receive the second infusion of cyclophosphamide in one month. The patient was seen two months after discharge in the hepatology clinic. He has been tolerating the anti-viral medications well with improvement of his HCV viral load to <12 copies/ml. However, he continues to have digital ulceration of his upper extremities alongside dry gangrenous lesions on his lower extremities. In addition, he continues to be hemodialysis dependent without improvement of his renal function.

## Discussion

PAN is a rare disease characterized by the necrotizing inflammation of medium-sized vessels [6]. The diagnosis of PAN relies on a combination of clinical manifestations, angiographic findings, and histopathological features of the biopsied tissue [1]. The mechanism of injury of PAN includes stenosis and rupture of inflamed visceral arteries leading to ischemic injury to the affected organs [1]. The most involved organs include the skin and peripheral nervous system [1]. GI involvement of PAN can manifest as severe inflammation and ischemia leading to acute abdomen [1]. Kidney involvement in PAN is due to inflammation of renal and interlobular arteries, and patients can present with hematuria, proteinuria, hypertension, and renal failure [1].

The association between HBV and PAN was first documented in 1970 [1]. However, with the development of vaccinations and new antiviral medications, the incidence of HBV-PAN has fallen below 5% [1]. Compared to HBV-PAN, HCV-PAN affects older patients and displays a more severe disease presentation, including more frequent fever, weight loss, and GI involvement [4]. HCV-PAN displays fewer cutaneous manifestations and a longer average period from viral infection to the development of vasculitis [7].

Distinguishing PAN from MC is often difficult because serum cryoglobulins are present in up to 60% of HCV patients [4]. Some of the differentiating factors between HCV-PAN and HCV-MC include more generalized manifestations, myalgia, GI tract involvement, and hypertension [4]. In addition, HCV-PAN displays a longer duration of viral infection to vasculitis compared to HCV-MC [4]. The CRP levels are also noted to be higher in HCV-PAN [4].

We share this interesting case as his presentation was congruent with the reported cases of HCV-PAN in the literature with the development of PAN more than 30 years after the diagnosis of HCV infection. Compared to HBV-PAN, our patient displayed a more accelerated and severe disease presentation with weight loss, nausea, vomiting, and cutaneous manifestation involving the bilateral shins. The renal involvement of our patient was severe leading to hemodialysis dependence in less than a week from disease manifestation. One unique factor about our patient was the absence of serum cryoglobulins, which are usually present in up to half of patients with HCV infection [4]. Unfortunately, there is no consensus on treatment for HCV-PAN, but some cases have been successfully treated with corticosteroids, cyclophosphamide, and antivirals [2].

## Conclusions

Our case highlights the importance of considering PAN in the differential diagnosis of patients with HCV infection who present with signs and symptoms of vasculitis. PAN-associated vasculitis in HCV-infected patients can present with more severe manifestations compared to HCV-MC. Therefore, prompt diagnosis is important in avoiding irreversible end-organ injuries to the patient.
